# Proteomic responses to progressive dehydration stress in leaves of chickpea seedlings

**DOI:** 10.1186/s12864-020-06930-2

**Published:** 2020-07-29

**Authors:** Saeedreza Vessal, Mohammad Arefian, Kadambot H. M. Siddique

**Affiliations:** 1 Research Center for Plant Sciences, Ferdowsi University of Mashhad, Mashhad, Iran; 2 Plant Biotechnology and Breeding Department, College of Agriculture, Ferdowsi University of Mashhad, Mashhad, Iran; 3 The UWA Institute of Agriculture, The University of Western Australia, Perth, WA 6001 Australia

**Keywords:** Chickpea, Comparative proteomics, Dehydration stress, Proline

## Abstract

**Background:**

Chickpea is an important food legume crop with high protein levels that is widely grown in rainfed areas prone to drought stress. Using an integrated approach, we describe the relative changes in some physiological parameters and the proteome of a drought-tolerant (MCC537, T) and drought-sensitive (MCC806, S) chickpea genotype.

**Results:**

Under progressive dehydration stress, the T genotype relied on a higher relative leaf water content after 3 and 5 d (69.7 and 49.3%) than the S genotype (59.7 and 40.3%) to maintain photosynthetic activities and improve endurance under stress. This may have been facilitated by greater proline accumulation in the T genotype than the S genotype (14.3 and 11.1 μmol g^− 1^ FW at 5 d, respectively). Moreover, the T genotype had less electrolyte leakage and lower malondialdehyde contents than the S genotype under dehydration stress, indicating greater membrane stability and thus greater dehydration tolerance. The proteomic analysis further confirmed that, in response to dehydration, the T genotype activated more proteins related to photosynthesis, stress response, protein synthesis and degradation, and gene transcription and signaling than the S genotype. Of the time-point dependent proteins, the largest difference in protein abundance occurred at 5 d, with 29 spots increasing in the T genotype and 30 spots decreasing in the S genotype. Some of the identified proteins—including RuBisCo, ATP synthase, carbonic anhydrase, psbP domain-containing protein, L-ascorbate peroxidase, 6-phosphogluconate dehydrogenase, elongation factor Tu, zinc metalloprotease FTSH 2, ribonucleoproteins and auxin-binding protein—may play a functional role in drought tolerance in chickpea.

**Conclusions:**

This study highlights the significance of genotype- and time-specific proteins associated with dehydration stress and identifies potential resources for molecular drought tolerance improvement in chickpea.

## Background

Plants are frequently exposed to abiotic stress such as dehydration in their natural habitats, which causes significant losses in yield and quality worldwide [1]. Climate changes are estimated to progressively increase the frequency, severity, and duration of drought periods [2]. To meet the needs of the growing world population, stabilizing or increasing yield potential under drought stress is imperative [3]. Plants respond to stress by reprogramming their proteins to ensure a steady-state of vital metabolic processes [4]. To this end, identifying novel proteins and responsive pathways involved in stress adaptation will provide new insights and enable direct genetic manipulations to improve climate-resistant crops.

Chickpea (*Cicer arietinum* L.) ranks third in terms of production and second in area, among the world’s food legume crops [5, 6]. Regardless of human dietary and health benefits, chickpea is effective at fixing atmospheric nitrogen and enriching soil fertility. Worldwide, chickpea yields more than 11 million tons from a cultivation area of ~ 12 million hectares [7]. Improvements in chickpea production have been slow, mainly because it is grown in rainfed areas where terminal drought is a major factor affecting its yield potential [8]. Although drought tolerance in chickpea is relatively higher than other cool-season legumes [9], seed yield losses due to drought varies from 30 to 100% [10, 11] depending on the type of drought and genotype.

Water stress reduces plant growth through physiological, biochemical, and molecular processes, including hormone induction, photosynthesis, respiration, kinase cascade signaling, ion uptake, carbohydrate and osmolyte metabolism, nitrogen assimilation, and amino acid metabolism. These processes can be analyzed using genes or their related proteins, but proteins play a critical role as they directly participate in plant responses to stress [12, 13]. Faster signal acquisition and better responses to water stress could be used to select or breed new plant genotypes with increased drought resistance. Hence, a precise and comparative analysis of the proteome in drought-sensitive and drought-tolerant plant genotypes is a fundamental step for understanding the mechanisms of stress adaptation physiology and water-use efficiency [14]. Wheat mutant plants exposed to drought stress had superior drought tolerance due to well-preserved leaf water balance, sustained membrane integrity, mobilized antioxidant defense and osmoregulation, and less affected photosynthetic activity [15–17].

To understand the molecular mechanisms responsible for dehydration stress, the events occurring in key functional molecules such as proteins need to be described. Although transcriptome profiling is a widely used technique to identify genes that are responsive to dehydration, there is far less evidence for proteins as functional products. Poor to moderate correlations between mRNA levels and the corresponding proteins have been reported in Arabidopsis [18]. Indeed, the role of genes in drought tolerance requires an understanding of their function at the protein level [19]. In recent years, several proteomic studies on dehydration have focused on the extracellular matrix [19, 20], cell nuclear [21–23], and seed germination [24] proteomes of chickpea seedlings. Proteome changes in chickpea have been investigated under other abiotic stresses, such as heat [25], salinity [26, 27], and abscisic acid [28]. The extracellular matrix (ECM) is a dynamic structure that includes important signaling components for front-line defense. An LC-ESI-MS/MS analysis identified dehydration-responsive proteins in chickpea’s ECM and revealed genotype-specific expression of proteins. It was also reported that cell wall restructuring and homeostasis of reactive oxygen species (ROS) are mainly influenced by a dehydration-adaptation mechanism [19, 20]. Among 4832 identified nuclear proteins of chickpea under drought stress, 299 unique phosphoproteins were involved in gene expression, protein degradation, and regulation of flowering time and the circadian clock [28]. Vessal et al. (2012) [24] investigated the germination of chickpea genotypes under limited water supply using 2-DE and MALDI-TOF/TOF LC-MS/MS analyses. Of 65 identified proteins, LEA and HSP proteins and proteins associated with ROS metabolism and the TCA cycle were identified as important pathways for coping with chickpea germination under water-limited conditions.

It is well-documented that chickpea growth and yields are highly significantly challenged by dehydration stress, with losses of up to 40–50% in crop productivity, mainly in areas lacking satisfactory and constant rainfall [29]. Chickpea is mainly grown in arid and semiarid areas, such as Iran, on stored soil moisture from the rainy season (winter or early spring); terminal or intermittent drought stress is a major constraint for productivity [8]. Consequently, breeding programs are needed to develop chickpea lines with higher water-use efficiency. We undertook a comparative analysis of drought-responsive proteins in a drought-tolerant and drought-sensitive chickpea genotype. Based on trait classifications reported by our colleagues [26, 30], MCC537 and MCC806 were considered as the most drought-tolerant and drought-sensitive genotypes, respectively, among 150 chickpea genotypes. Some key physiological and biochemical parameters have been evaluated in these contrasting genotypes, including root and shoot fresh and dry weights, relative water content, proline, chlorophyll, carotenoid and MDA contents, antioxidant activity (CAT, GR, SOD, APX and DPPH), and sodium and potassium concentrations in roots and shoots. In the current study, along with some physiological assessments, proteome alterations in these genotypes under drought stress were explored using two-dimensional gel electrophoresis coupled with mass spectrometry to identify 34 differentially modulated spots.

To our knowledge, there are no reports on total proteome changes in chickpea leaves exposed to dehydration stress. The lack of sufficient genomic information at the functional protein level prompted us to evaluate early physiological responses and undertake a comprehensive proteomic analysis of dehydration stress responses in two extreme chickpea genotypes. We identified some novel dehydration stress-responsive leaf proteins, differentially expressed in chickpea seedlings exposed to a progressive stress situation.

## Methods

### Plant material and stress treatment

We used MCC537 (T) and MCC806 (S) as drought-tolerant and drought-sensitive chickpea genotypes, respectively, based on previous reports [26, 30]. Seeds of genotypes were acquired from the Research Center for Plant Sciences, Ferdowsi University of Mashhad, Iran. The seeds were surface sterilized in 3% (w/v) sodium hypochlorite and 70% ethanol for 1 min, followed by five washes with sterile water. The sterilized seeds were germinated on moistened Whatman filter paper in 10 cm diameter Petri dishes at 25 ± 1 °C for 48 h in a germinator. Four uniformly germinated seeds were transferred to 2 L pots containing loam soil and sand (2:1, w/w) in a greenhouse set at 28/20 ± 3 °C (day/night), 60 ± 5% relative humidity, and a ~ 16 h photoperiod. After 1 week, each pot was thinned to two seedlings and irrigated with 150 mL of water daily to maintain soil water content at ~ 40%. At 28 days after sowing, the seedlings in the stress treatment were subjected to gradual, progressive dehydration by withdrawing water and allowing evapotranspiration; the control plants were watered normally. The middle leaves of each seedling were harvested after 1, 3, and 5 d of dehydration exposure for each genotype. All treatments were conducted in triplicate.

### Physiological analysis

The relative water content (RWC) of leaf tissue was determined using the following equation: RWC (%) = [(FW − DW) / (TW − DW)] × 100, where FW is fresh weight, DW is dry weight, and TW is turgid weight [31].

Electrolyte leakage (EL) was determined by estimating the leaching of ions from leaves into distilled water [32]. Briefly, 0.1 g leaves were placed in 10 mL distilled water in a Falcon tube in two sets, the first at 40 °C for 30 min and the second at 100 °C for 10 min in a water bath, before recording conductivity using EC meter (C1 and C2, respectively). The EL (%) was calculated as (C1/C2) × 100.

Free proline content was determined using a colorimetric assay using the method developed by [33]. Leaf tissue (0.2 g) was homogenized in 4 mL 3% aqueous sulfosalicylic acid and then centrifuged at 9000 *g* for 10 min. The supernatant was mixed with ninhydrin and glacial acetic acid in equal ratio, then boiled at 100 °C for 1 h and the absorbance read at 520 nm. The proline concentration was measured using the standard curve and expressed as μmol proline g^− 1^ FW.

Membrane lipid peroxidation was determined from malondialdehyde (MDA) accumulation using the procedure of Heath and Packer (1968) [34]. Fresh leaf tissue (0.2 g) was ground in 0.1% trichloroacetic acid (TCA) and centrifuged at 9000 *g* for 10 min. The supernatant was mixed with 20% TCA, containing 0.5% thiobarbituric acid in a 1:4 (v/v) ratio and heated at 90 °C for 30 min. Absorbance (A) was recorded at 532 mm and 600 nm, and MDA calculated according to MDA (μmol g^− 1^ FW) = (A_532_ – A_600_) / 1.55 × 10^− 5^ M cm × b.

### Sample preparation and two-dimensional electrophoresis (2-DE)

To identify proteins involved in drought tolerance, leaf samples were ground in liquid nitrogen to a fine powder and total protein extracts prepared using the method of Goggin et al. (2011) [35]. Briefly, the finely ground leaf samples (1 g) were suspended in extraction buffer containing 8 M urea, 2% (v/v) Triton X-100, 25 mM DTT, and 2% (w/v) CHAPS and held at − 4 °C for 15 min. After centrifugation at 12,000 *g* for 10 min, the pellets were air-dried before being dissolved in 9 mL chilled acetone containing 3% TCA and incubated at − 80 °C for 1 h. Soluble chlorophyll and other impurities were removed by centrifugation at 14,000 *g* for 30 min; the pelleted protein was dissolved in minimal isoelectric focusing (IEF) buffer contained 8 M urea, 4% (w/v) CHAPS, 65 mM DTT, and 2% (v/v) immobilized pH gradient (IPG) buffer pH 4–7. The protein concentration of each sample was determined using the method of MM Bradford [36].

The solubilized proteins (400 μg) were used to rehydrate 17 cm IPG strips at pH 4–7 (BioRad). The proteins were covered with mineral oil to avoid urea crystallization and rehydrated for 16 h at room temperature. The strips were subjected to electrophoresis for 38 kV.h using the PROTEAN IEF Cell System (BioRad, USA) before equilibration in 15 mL equilibration solution (6 M urea, 50 mM Tris pH 8.8, 30% [v/v] glycerol, 2% [w/v] SDS and 0.002% [w/v] bromophenol blue) containing 65 mM DTT, and 135 mM iodoacetamide for 15 min for each equilibration solution. Protein separation in the second dimension was performed with SDS-PAGE gels containing 12.5% (w/v) polyacrylamide on PROTEAN II MultiCell (BioRad, USA).

### Gel staining, imaging, and data analysis

The separated proteins were visualized using 0.12% (w/v) Coomassie Brilliant Blue G-250 staining solution containing 10% orthophosphoric acid (v/v) and 10% ammonium sulfate (w/v) as described by Candiano et al. (2004) [37]. The stained gels were scanned using a GS-800 Calibrated Densitometer (BioRad) at a resolution of 600 dpi. Gel images were analyzed using ImageMaster™ 2D Platinum Version 6.0 (GE Healthcare Bio-Science). The experimental molecular mass (*M*_r_) and isoelectric point (p*I*) of the proteins were determined using standard protein markers (Sigma, USA) and the relative migration of protein spots on IPG strips, respectively. The analysis was based on percent volumes, and significant differences (*p* ≤ 0.05) in the expression of the spot were calculated using one-way analysis of variance and Duncan’s multiple range test if the mean abundance varied more than two-fold.

### Protein identification

Protein spots were identified by liquid chromatography coupled with tandem mass spectrometry (LC-MS/MS) analysis, as described by Bringans et al. (2008) [38], at Proteomics International, Nedlands, Western Australia, using an Agilent 1260 Infinity HPLC system (Agilent) coupled to an Agilent 1260 Chipcube Nanospray interface (Agilent) on an Agilent 6540 mass spectrometer (Agilent). The raw spectral data were processed into MASCOT Generic File format, and the MS/MS ion search was performed using MASCOT (http://www.matrixscience.com) to search the NCBInr database and using Viridiplantae (green plants) as the taxonomy. The parameters for the search were: an MS/MS tolerance of ±0.2 Da, one missed cleavage site, enzyme of trypsin, variable modifications of carbamidomethyl, peptide tolerance of ±0.2 Da, peptide charge of 2+ 3+ 4+, monoisotopic, and ESITRAP instrument. The score threshold to achieve *p* < 0.05 was set by the MASCOT algorithm, which is based on the size of the database used in the search. In the case of multiple significant hits for a protein, only the highest scoring hit is listed in Table 1. Details on the theoretical and experimental molecular weights and isoelectric points, percent coverage, and accession number for proteins identified with a single peptide are listed in Table 1. The function of the identified proteins was analyzed in terms of the metabolic role of the identified protein in response to stress by searching the NCBI and UniProt databases and literature reviews [39].

### Statistical analysis

The results are presented as means ± SE. To identify statistically significant differences between means, one-way ANOVA and Duncan’s multiple range test were performed with a 5% level of significance using SPSS software (ver. 24).

## Results

### Dehydration stress responses of chickpea seedlings at the physiological level

**Fig. 1:**
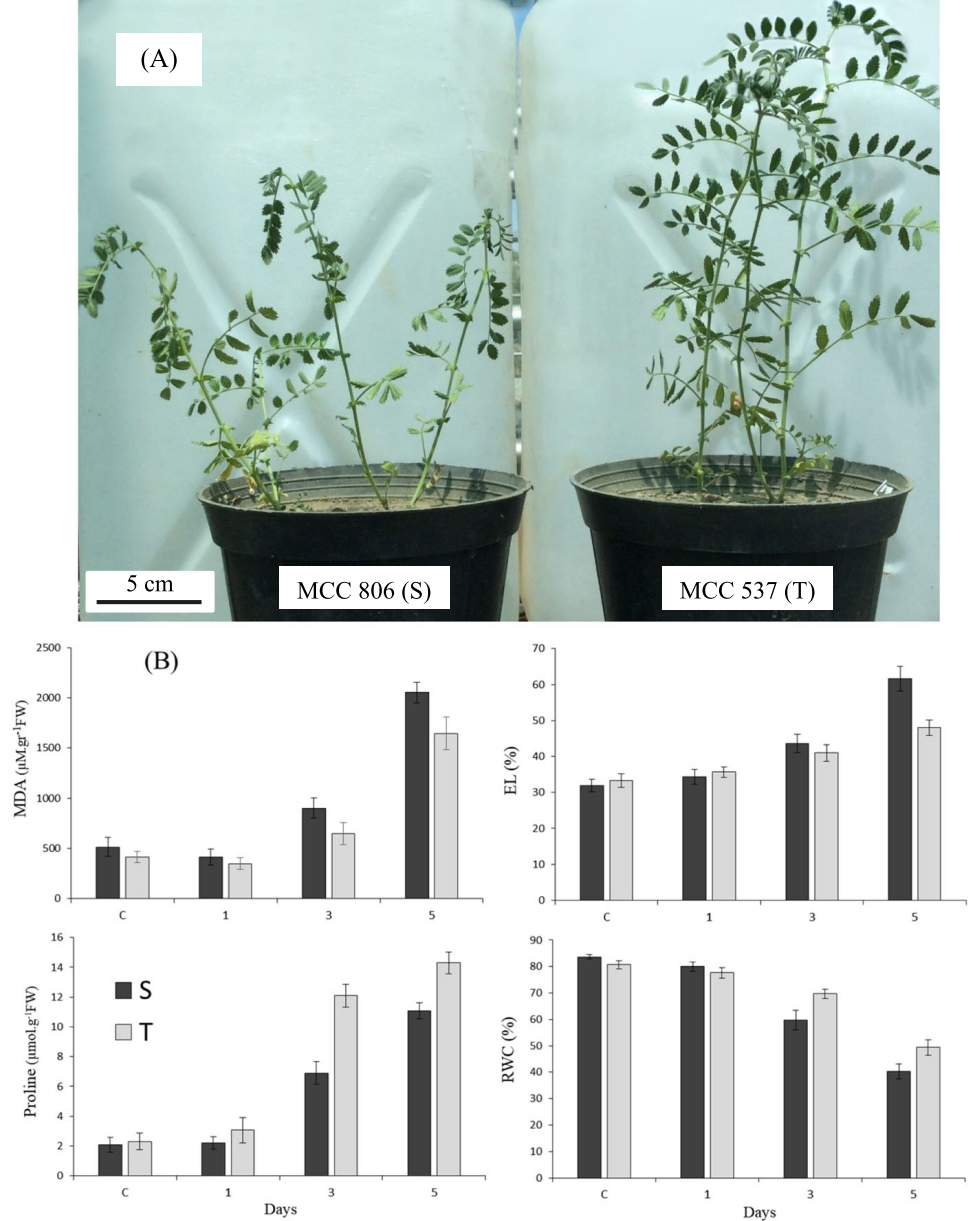
**a** Morphological response of 28-day-old chickpea seedlings to progressive dehydration stress for five days. **b** Physiological changes in seedlings of drought-tolerant (MCC537, T) and drought-sensitive (MCC806, S) chickpea genotypes after 1, 3 and 5 days of dehydration treatment, relative to the control (C), including malondialdehyde (MDA), electrolyte leakage (EL), proline, and relative water content (RWC) of leaves. Error bars indicate the standard error of three biological replicates

Physiological responses to dehydration stress were monitored in 28-day-old seedlings of the selected tolerant (T) and sensitive (S) chickpea genotype to identify differences in drought resistance. Changes in EL, as a biomarker of membrane integrity [40], were measured over 5 days of dehydration stress. The EL index increased moderately from 1 d to 3 d of stress in both genotypes, relative to the control, with a significant increase from 3 d to 5 d of stress, especially in the S genotype (Fig. 1b). Malondialdehyde levels declined after the first day of stress, relative to the controls, then increased significantly up to 5 d of dehydration stress, more so in the S genotype. After 5 days of dehydration stress, leaf RWC had declined by 43 and 31% in the S and T genotypes, respectively, relative to their respective controls, and was accompanied by increasing proline contents. After 5 d of dehydration stress, the S and T genotypes had 5.3- and 6.2-fold higher proline contents, respectively, than the controls (Fig. 1b).

### Proteome changes in response to dehydration stress in two chickpea genotypes

**Fig. 2:**
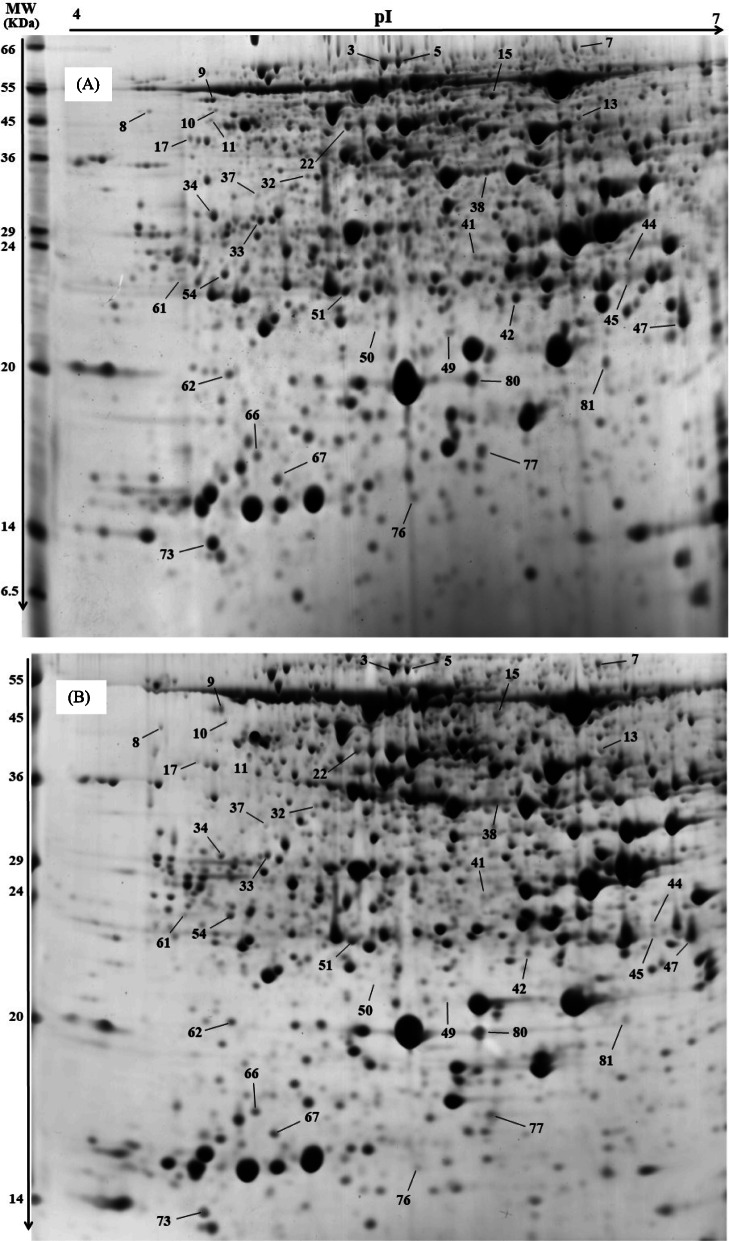
Representative 2-D photos of chickpea leaf proteins stained by Coomassie blue in the **a** drought-tolerant (MCC537, T), and **b** drought-sensitive (MCC806, S) genotypes. First dimension: 17 cm IEF strips pH 4–7 linear, second dimension: SDS-PAGE containing 12.5% (w/v) polyacrylamide. Lines indicate differentially regulated protein spots subjected to LC-MS/MS analysis

**Fig. 3:**
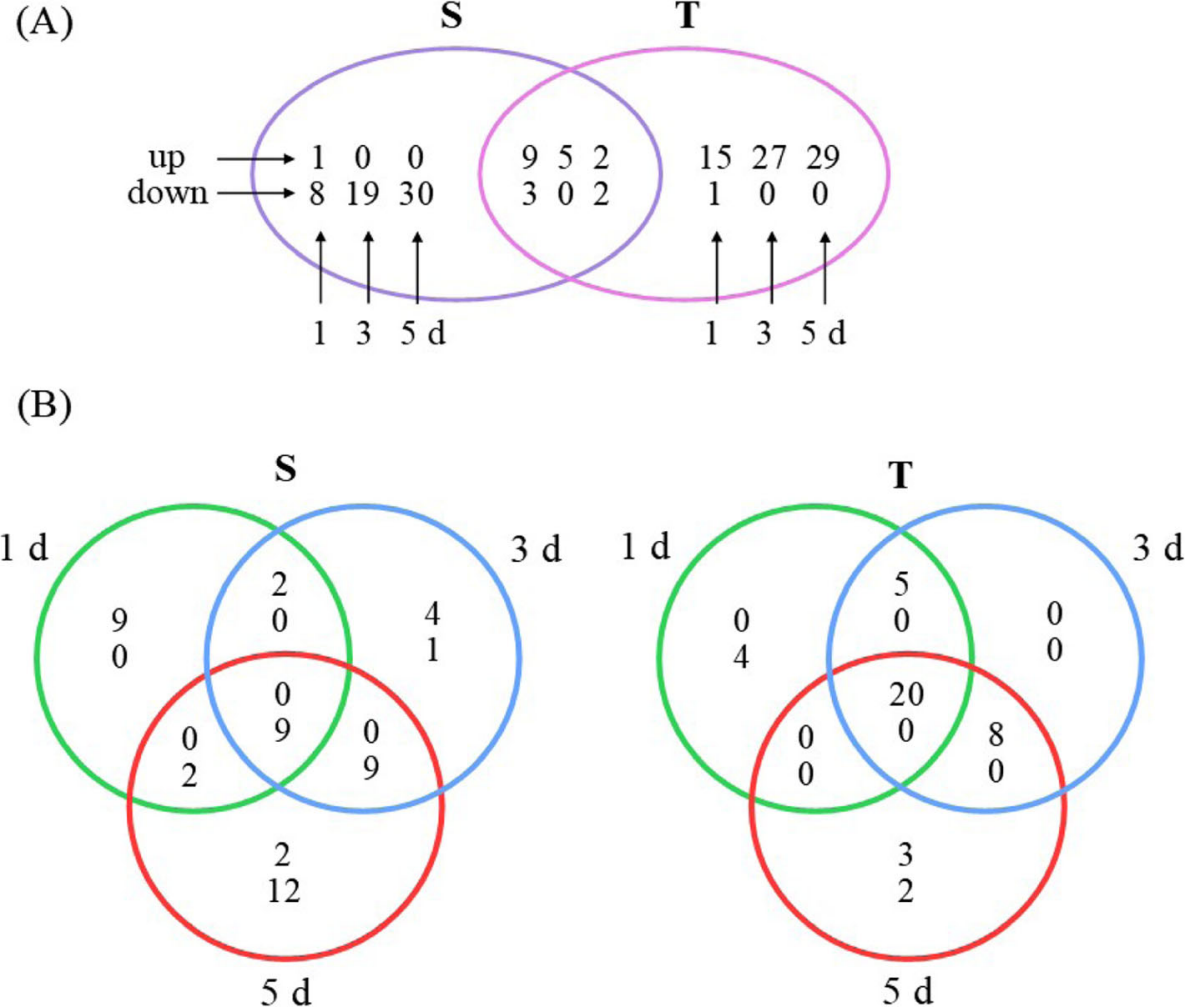
Venn diagram comparing differentially expressed proteins (DEPs) with controls among sensitive (S) and tolerant (T) genotypes of chickpea after 1, 3, and 5 days (d) of dehydration stress. **a** Number of genotype-dependent DEPs at each time-point (or shared between). **b** Number of time-point-dependent DEPs (or shared between) in each genotype. The up and down arrows indicate the number of increased and decreased DEPs, respectively, relative to the respective controls

This study detected 1184 reproducible protein spots, of which 237 had significant changes in abundance during the stress, relative to the controls. Protein spots with absolute variation (≥1.5-fold, *p* < 0.05) in the quantitative image analysis were considered to have changed significantly after dehydration stress and analyzed further. Thirty-four proteins were characterized as differentially expressed proteins (DEPs) between the two genotypes in response to dehydration stress (Fig. 2). The T genotype generally had more up-regulated proteins than the S genotype at each time-point (Fig. 3).

**Fig. 4:**
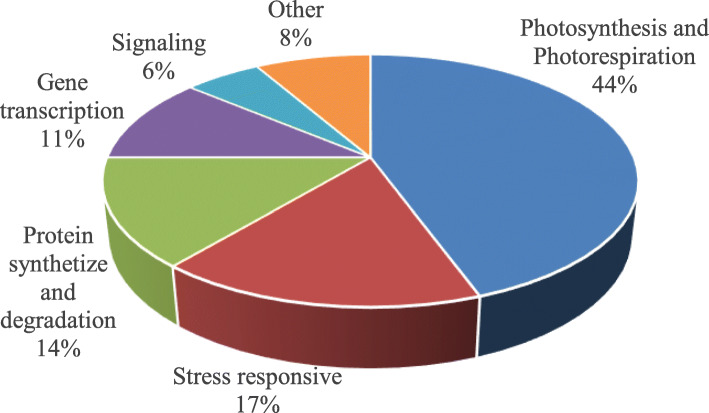
Functional classification of stress-responsive proteins in chickpea (*n* = 34)

Genotype- and stress time-point dependent DEP spots were examined in a Venn diagram (Fig. 4a). The two genotypes differed in protein expression in response to dehydration; the T genotype responded to dehydration by increasing up-regulated spot proteins (29 spots at 5 d), the S genotype had more down-regulated proteins (30 spots at 5 d). Both genotypes had similar expression patterns of some spots in response to dehydration, with 16 up-regulated spots and five down-regulated spots (Fig. 3a).

Figure 3b identifies the DEPs shared by, or specific to, each time-point. Both genotypes had the largest difference in abundance after 5 d of stress. Across all time-points, the T genotype shared 20 up-regulated spots, while the S genotype only shared nine down-regulated spots. Of the time-point dependent spots, the S genotype uniquely regulated nine, five, and 14 spots, and T genotype regulated four, zero, and five spots after 1, 3, and 5 d of dehydration stress, respectively (Fig. 3b).

**Fig. 5:**
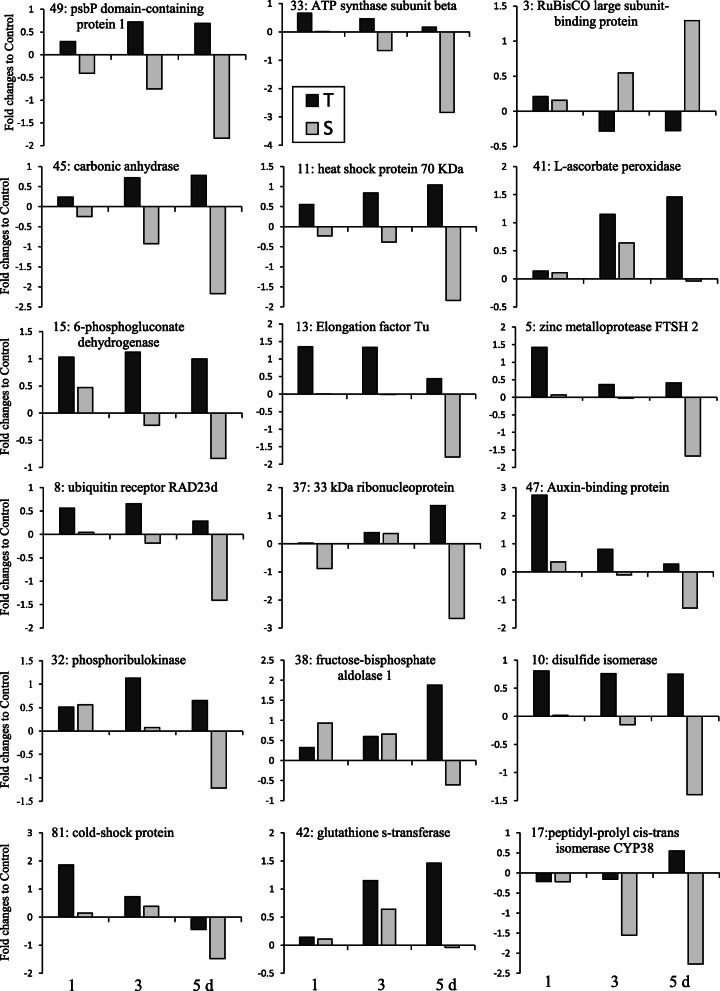
Fold-changes in the abundance of some important dehydration-responsive proteins, relative to the control, in terms of function and the rate of differential expression between two chickpea genotypes (MCC537, T, and MCC806, S) after 1, 3 and 5 days of dehydration treatments. Numbers before the name of each protein correspond with the spot numbers specified in Table 1 and Fig. 2

Table 1 summarizes the proteins identified by LC-MS/MS, including names (where applicable), MASCOT score, percent coverage, theoretical/experimental p*I* and *Mr*, and accession numbers. Most of the identified proteins were strictly matched to chickpea (*Cicer arietinum* L.) with a high score and decent sequence coverage. The proteins were grouped into six categories by putative function. Most were involved in photosynthesis (44%), stress response (17%), and protein synthesis and degradation (14%) (Fig. 4). Of the identified proteins (34 protein spots), some were critical for functional responses to stress and also had differential expression between the two genotypes, including ATP synthase, RuBisCo, psbP domain-containing protein, carbonic anhydrase, L-ascorbate peroxidase, elongation factor Tu and 6-phosphogluconate dehydrogenase (Fig. 5). In most of these critical proteins, differences in abundance increased with stress duration.

**Table 1:** Proteins from drought-treated chickpea with ≥1.5-fold alteration, relative to control values, identified by LC-MS/MS analysis and Mascot database search

Spot no.^a^	Protein identity	Species^b^	Coverage (%)^c^	MASCOT score^d^	TM_**r**_/EM_**r**_^e^	Tp***I***/Ep***I***^f^	Acc. No.^g^
	**Photosynthesis and photorespiration**						
3	RuBisCO large subunit-binding protein subunit beta	*Cicer arietinum*	44	1137	62.9/59.2	5.85/5.53	XP_012567814.1
59	Probable ribose-5-phosphate isomerase 3, chloroplastic	*Cicer arietinum*	29	654	30.5/21.7	6.00/4.88	XP_004494688.1
66	Ribulose-bisphosphate carboxylase large subunit, partial	*Salix bebbiana*	18	55	23.5/14.5	5.61/4.94	AIG57943.1
7	Glycine dehydrogenase (decarboxylating), mitochondrial	*Cicer arietinum*	26	633	115.3/60.2	7.99/6.43	XP_004498896.2
33	ATP synthase subunit beta, chloroplastic	*Cicer arietinum*	39	585	52.9/28.7	5.16/4.99	B5LMK9
56	ATP synthase subunit alpha, chloroplastic	*Cicer arietinum*	54	321	19.4/21.5	5.05/5.16	B5LMN1
38	Fructose-bisphosphate aldolase 1, chloroplastic	*Cicer arietinum*	21	1376	43.2/33.5	6.28/5.98	XP_004507507.1
45	Carbonic anhydrase, chloroplastic isoform X2	*Cicer arietinum*	19	409	35.8/22.3	6.61/6.66	XP_004489275.1
49	psbP domain-containing protein 1, chloroplastic	*Cicer arietinum*	65	643	28.6/20.5	8.89/5.79	XP_004494530.1
22	ATP synthase beta subunit, partial (chloroplast)	*Eucryphia lucida*	50	655	51.8/40.2	5.20/5.38	AAK72764.1
32	Phosphoribulokinase	*Erythranthe guttata*	13	148	45.5/33.1	5.86/5.20	A0A022QP63
50	Chlorophyll a-b binding protein 3, chloroplastic	*Cicer arietinum*	22	511	29.4/20.7	6.32/5.56	XP_004491629.1
57	Oxygen-evolving enhancer protein 2, chloroplastic	*Cicer arietinum*	29	721	28.7/19.0	5.65/5.55	XP_004499534.1
62	Oxygen-evolving enhancer protein 1, chloroplastic	*Cicer arietinum*	45	679	34.9/19.0	6.24/4.83	XP_004509219.1
67	Oxygen-evolving enhancer protein 2, chloroplastic	*Cicer arietinum*	30	643	28.7/14.2	6.90/5.02	XP_004499534.1
73	Oxygen-evolving enhancer protein 2, chloroplastic	*Cicer arietinum*	16	117	28.7/9.25	6.90/4.72	XP_004499534.1
	**Stress responsive**						
11	Heat shock protein 70	*Chrysanthemum morifolium*	13	265	70.8/42.2	5.12/4.86	A0A0A1HAD2
41	L-ascorbate peroxidase, cytosolic	*Cicer arietinum*	37	243	27.1/23.2	5.65/ 5.94	XP_004505943.1
51	L-ascorbate peroxidase, cytosolic	*Cicer arietinum*	57	781	27.1/20.75	5.65/5.36	XP_004505943.1
42	Glutathione s-transferase	*Cicer arietinum*	34	270	25.6/21.8	6.04/6.12	A0A0X9LEN0
77	Superoxide dismutase [Cu-Zn]	*Solanum chacoense*	15	1057	22.4/14.5	6.08/5.96	A0A0V0HK97
81	Cold shock protein	*Cicer arietinum*	31	1497	19.2/18.5	6.29/6.55	A0A088FZS5
	**Protein synthesis and degradation**						
13	Elongation factor Tu, mitochondrial	*Cicer arietinum*	30	272	49.1/40.2	6.58/6.44	XP_004493639.2
17	Peptidyl-prolyl cis-trans isomerase CYP38, chloroplastic	*Cicer arietinum*	9	82	50.1/39.5	5.12/4.68	XP_004489294.1
10	Uncharacterized protein LOC101507383, partial, homoluguse with protein disulfide isomerase (query coverage 99%)	*Cicer arietinum*	15	152	42.7/46.2	4.83/4.81	XP_004495295.1
5	ATP-dependent zinc metalloprotease FTSH 2	*Cicer arietinum*	7	88	74.7/62.7	5.60/5.34	XP_004504668.1
8	Ubiquitin receptor RAD23d-like	*Cicer arietinum*	10	55	40.3/33.7	4.39/4.53	XP_004489115.1
	**Gene transcription**						
37	33 kDa ribonucleoprotein, chloroplastic	*Glycine soja*	3	66	30.4/31.5	8.67/4.98	A0A0B2SU82
61	28 kDa ribonucleoprotein, chloroplastic	*Cicer arietinum*	26	642	29.8/24.5	4.74/4.64	XP_012570426.1
54	29 kDa ribonucleoprotein A, chloroplastic	*Cicer arietinum*	16	110	30.7/23.2	5.36/4.83	XP_004497514.1
76	U3 small nucleolar ribonucleoprotein protein MPP10-like isoform	*Malus domestica*	1	61	62.3/13.2	4.5/5.6	XP_008367517.1
	**Signaling**						
47	Auxin-binding protein ABP19a-like	*Cicer arietinum*	28	569	21.9/20.7	6.95/6.91	XP_004513480.1
80	Low molecular weight phosphotyrosine protein phosphatase	*Cicer arietinum*	12	287	27.1/17.7	7.62/5.9	XP_004506147.1
	**Other**						
44	Tropinone reductase homolog At5g06060-like	*Cicer arietinum*	27	250	28.5/22.5	6.43/6.66	XP_004492175.1
9	COBW domain-containing protein	*Cicer arietinum*	39	265	50.8/48.5	5.81/4.79	XP_004497313.1
15	6-phosphogluconate dehydrogenase, decarboxylating 3	*Cicer arietinum*	43	524	53.6/47.2	5.88/5.99	XP_004491970.2

^a^ Numbering corresponds to the 2-DE gel in Fig. 2a

^b^ Species name that the identified proteins is obtained via the MASCOT software from the NCBI database

^c^ Sequence coverage percentage of assigned peptides to the predicted protein

^d^ Score probability for the entire protein, obtained by Mascot search engine

^e^ TMr and EMr are theoretical molecular mass and experimental molecular mass, respectively

^f^ Tp*I* and Ep*I* are theoretical isoelectric point and experimental isoelectric point, respectively

^g^ Corresponding gene identification number in GenBank

## Discussion

Plants reduce water loss under drought stress by closing stomata, which reduces CO_2_ uptake and decreases photosynthetic efficiency. In this condition, excess energy excitation occurs, which subsequently produces ROS and methylglyoxal that negatively affect protein, lipid, and DNA function. To cope with this, plants resort to adaptive strategies at the physiological and molecular levels, including repair and detoxification, especially through specific-protein functions [41]. These events often vary among genotypes of a specific plant species—these differences can offer valuable perceptions for developing strategies for crop improvement. Most of the research on chickpea drought stress has focused on physiological, genome, and transcript methods [42–46]. However, as drought stress impacts actively on protein-related processes such as synthesis and degradation, proteomic studies of drought stress could significantly improve our insight into its repercussions at a functional level. Therefore, the current study characterized proteins to reveal genotype-specific variations in the leaf proteome in response to drought stress in contrasting chickpea genotypes, which led to the final characterization of 34 drought stress-induced DEPs. The largest difference in protein abundance across all time-points occurred after 5 d of stress in both genotypes, when 29 protein spots were up-regulated in the T genotype. Comparative proteomics revealed genotype-specific and drought stress expression of proteins involved in a variety of cellular functions, which may offer some likely candidates for increasing tolerance to drought stress in chickpea. The findings suggest that the up-regulated proteins, especially those in the T genotype at 5 d including ATP synthase subunits, psbP domain-containing protein, carbonic anhydrase, RuBisCo subunits, elongation factor Tu and disulfide isomerase., are associated with important processes such as photosynthesis, protein metabolism, and signaling, which included.

The main source of plant biomass is photosynthesis, which influences potential crop yield and is often sensitive to drought stress; hence, it is a priority for crop breeding programs. In the leaf quantitative proteomic analyses, 44% of the proteins whose abundance changed significantly in response to dehydration were associated with photosynthesis and photorespiration (Fig. 4 and Table 1). Diverse changes in these proteins have been reported in other plant species under salt stress [47] and heat stress [25]. Six of the identified proteins are involved in light-harvesting complexes (Table 1): psbP domain-containing protein 1 (spot 49), chlorophyll a-b binding protein 3 (spot 50), and oxygen-evolving enhancer protein1, 2 and 3 (spots 62, 57, 67 and 73).

The superfamily of chlorophyll a-b binding proteins is associated with photosystem I (PSI) or photosystem II (PSII) [48]. In this study, the psbP domain-containing protein (Fig. 5; spot 49) and chlorophyll a-b binding protein (spot 50) were up-regulated in the T genotype, possibly because dehydration stress increased photosynthesis more in the T genotype than the S genotype. These proteins provide energy to reduce NADP^+^ to NADPH in PSI. The accumulation of proteins associated with light-harvesting complexes in response to drought stress may prevent or reduce light stress-induced damage [49]. Differences between genotypes in the expression of the chlorophyll a-b binding protein likely reflect the redox state of plastoquinone [50]; however, this mechanism should be explored to elucidate its role in chickpea drought tolerance. Consistent with our findings, chlorophyll a-b binding proteins were expressed in response to stress in *Arabidopsis* [51] and salt-treated *Spinacia oleracea* [52], and are required for efficient repair of PSII photodamage.

The ATP synthase function is directly related to photosynthesis as it transfers protons through thylakoid membranes. The response of ATP synthase to salinity depends on plant species, genotype, and the duration and severity of stress [53]. All ATP synthase subunits alpha and beta detected in this study were up-regulated in response to dehydration stress in the T genotype (Fig. 5; spot 33). After 5 days of dehydration stress, the subunits of the ATP synthase complex in the S genotype declined ~ 3-fold, indicating high regulation of the whole machinery. ATP synthase activity facilitates non-photochemical quenching, a process that protects the photosynthetic apparatus from light-damage; reductions in its activity have been reported in other species under stressful conditions [54].

RuBisCo (EC 4.1.1.39) accounts for a major portion of soluble leaf proteins (about 30–70 g/100 g) and is a key player in photosynthesis. RuBisCo catalyzes carbon metabolism at an early stage of CO_2_ fixation in photosynthetic eukaryotes and plays a role in phosphoglycolate synthesis, which is re-circulated by photorespiration. Photorespiration drains the pool of sugar substrate (RuBP) and reduces the efficiency of carbon fixation by up to 50%. Thus, the key role of RuBisCo is to suppress oxygenation and improve carboxylation, which is necessary for some important characteristics such as biomass, plant height, and yield in drought-tolerant crop genotypes [55]. In this study, 3 d and 5 d of dehydration stress induced RuBisCo subunits in the T genotype (Fig. 5; spot 3). Other enzymes involved in the Calvin cycle, including carbonic anhydrase (Fig. 5; spot 45), phosphoribulokinase (spot 32), and fructose-bisphosphate aldolase 1 (spot 38), increased in abundance in the T genotype, relative to the control and the S genotype. These proteins have been associated with abiotic stress tolerance in chickpea, including heat [25] and drought [19, 20] stress. In line with earlier reports, our results show that the accumulation of osmo-protectants could be another strategy embraced by the T genotype to cope with dehydration stress by retaining cell turgor pressure [56] and thus maintaining RWC. Moreover, proline can assist in the maintenance of photosynthetic efficiency, cellular redox potential, and antioxidant free radical amounts in thylakoid membranes [45]. The greater stress-dependent proline level and RWC in the T genotype relative to the S genotype (Fig. 1b) suggests that the T genotype can moderate its proline content for cell defense and osmotic adjustment to cope with dehydration stress. Other studies have reported proline accumulation in chickpea in response to drought and salinity, especially in tolerant genotypes [17, 46]. The correlation between enhanced levels of photosynthetic enzymes [55] and proline content may have increased photosynthesis, biomass, and yield more in the T genotype than the S genotype under dehydration stress [16].

In this study, six proteins were specifically involved in the dehydration stress response: heat- and cold-shock proteins (spots 11 and 81), L-ascorbate peroxidase (spots 41 and 51), superoxide dismutase (spot 77), and glutathione s-transferase (spot 42) (Table 1). Drought stress causes protein dysfunction by denaturation and aggregation [57]. Hence, many HSPs are up-regulated under stress to stabilize proteins and membranes and refold proteins [58]. The induction of HSP70 in the T genotype, relative to the control and the S genotype (Fig. 5, spot 11), suggests that protein stabilization and refolding may have increased to cope with dehydration stress. The induction of HSPs has been reported in salt- and water-stressed rice [59], potato [60], and soybean [61].

The cold-shock domain protein is a highly conserved nucleic acid binding domain that may act as an RNA-chaperone in the regulation of translation [62], and has been induced in the leaves of rice [63] and roots of wheat [64] under salt stress. It is possible that higher expression of the cold-shock protein (Fig. 5, spot 81) in the T genotype than the S genotype during the early stages of dehydration stress facilitated the translation process by removing secondary structures of mRNA and regulating gene expression by dsDNA interaction.

Production of ROS due to stress leads to membrane loss, accompanied by ion leakage, that is harmful to chlorophyll and the photosynthetic apparatus, and diminishes plant productivity [65]. In the present study, a considerable increase of antioxidant and detoxification enzymes in the T genotype was reflected in higher RWC, reduced EL, and less MDA, suggesting that protection against oxidative stress damage is a principal endurance mechanism in chickpea. In contrast, drought stress had a more negative effect on the S genotype, with high MDA content and increased EL, which has been associated with enhanced protein degradation and lipid peroxidation [66]. The slight initial decline in MDA content in both genotypes could be due to an increase in unsaturated fatty acids during the first day of stress [42, 43]. The T genotype had higher levels of antioxidant enzymes than the S genotype, mostly after 3 d and 5 d of dehydration stress (Fig. 5, spot 41). Redox homeostasis is maintained in plants by antioxidant enzymes and low molecular-weight osmolytes [67]. Superoxide dismutase (SOD) converts the potentially harmful superoxide radical (O^−^_2_) to molecular oxygen and H_2_O_2_, thus playing an effective role in cell defense and rescue [68].

Glutathione s-transferase is an important detoxification enzyme in the GPX/GST pathway, removing H_2_O_2_ [69]. The GPX/GST pathway, as an alternative mechanism for removing H_2_O_2_, was activated after 5 d of stress, especially in the T genotype (1.6-fold), to compensate for reduced expression of ascorbate peroxidase (Fig. 5, spot 42). Time- and genotype-dependent regulation of antioxidants were observed in this study. Similarly, Salekdeh et al. (2002) [70] reported a diverse abundance of antioxidants in rice under drought stress, indicating that their functional state is dependent on subcellular location, stress conditions, and genotype. Consistent with our results, tolerant genotypes of soybean [71, 72] and canola [73] had higher expression of superoxide dismutase and ascorbate peroxidase than susceptible genotypes. Thus, it appears that these antioxidants play an essential role in the drought tolerance of the T genotype. NADPH can be generated through the oxidative pentose pathway and used to prevent oxidative stress [74]. Salt stress induced 6-phosphogluconate dehydrogenase in barley leaves [74] and rice shoots [75]. An increased abundance of 6-phosphogluconate dehydrogenase (Fig. 5, spot 15) in the T genotype may have increased NADPH production to supply the required energy for drought tolerance. Based on our results, it is likely that the T genotype has higher membrane integrity than the S genotype—as evidenced by its reduced LA and MDA content, higher RWC, and abundance of heat- and cold-shock proteins, L-ascorbate peroxidase, superoxide dismutase, and glutathione s-transferase—thereby reducing dehydration stress damage to membranes [15, 17] and possibly increasing water-use efficiency.

The protein biogenesis and degradation group accounted for 14% of the DEPs in the stressed seedlings (Fig. 4). Dehydration stress significantly increased the elongation factor Tu (Fig. 5, spot 13) level in the T genotype after 1 d and 3 d but significantly decreased it in the S genotype at 5 d (Fig. 5). Elongation factors contribute to the initiation and elongation of newly growing peptide chains [76], which may explain why the enhanced biosynthesis or repair of drought-stressed proteins. In this study, the abundance of peptidyl-prolyl cis-trans isomerase CYP38 (Fig. 5; spot 17) and disulfide isomerase (Fig. 5; spot 10) increased in the T genotype, indicating its greater ability for protein biogenesis, folding and stability than the S genotype.

Proteases have essential roles in plants by maintaining protein quality control and degrading specific sets of damaged proteins and other unstructured peptides under stress [77]. The levels of ATP-dependent zinc metalloprotease FTSH 2 (spot 5) and ubiquitin receptor RAD23d (spot 8) decreased significantly in the S genotype at 5 d (Fig. 5). The reduction in degrading proteins at specific times in this experiment may suggest a reduction in proteolysis of peculiar proteins that may be needed during drought stress. However, the observed differential regulation of distinct components in the protein biogenesis and degradation machinery suggests that a complicated mechanism is involved in controlling protein metabolism under drought stress that depends on time and genotype.

Four identified proteins—ribonucleoproteins (spots 37, 61, 54, and 76)—were classified as gene transcription proteins (Table 1) that are involved in RNA processing [78]. These proteins, which accumulated more in the T genotype than the S genotype under dehydration stress (Fig. 5, spot 37), are a regulatory factor in the response of plants to biotic and abiotic stresses through the translation of defense-related genes in the chloroplast [79].

This study identified two dehydration stress-induced proteins involved in signaling—auxin-binding protein ABP19a-like (spot 47) and low molecular-weight phosphotyrosine protein phosphatase (spot 80)—that increased after 1 d of stress, especially in the T genotype, but decreased after 5 d of stress, especially in the S genotype (Fig. 5; spot 47). Auxin is a fundamental hormonal signaling agent in cells, which functions as an integrator for other phytohormones to control plant growth under environmental stress [80]. Our results suggest that dehydration stress enhances the auxin signal transduction pathway in the leaves of chickpea seedlings by increasing the amount of auxin-binding protein during the initial stages of dehydration stress in both genotypes, especially the T genotype, which would alter many metabolic pathways and cellular processes to cope with dehydration stress. This should be validated in further experiments.

## Conclusion

To identify drought-responsive proteins in chickpea, two contrasting genotypes in terms of drought tolerance were compared using some physiological parameters and a proteomic approach. The proteome alterations suggest that the up-regulated proteins, especially those in the T genotype at 5 d, are associated with photosynthesis, protein metabolism, and signaling, and included ATP synthase subunits, psbP domain-containing protein, carbonic anhydrase, RuBisCo subunits, elongation factor Tu and disulfide isomerase. These proteins might contribute to alleviating the harmful effect of dehydration stress on chlorophyll biosynthesis, photosynthesis, energy synthesis, and gene expression in chickpea leaves. The major protein differences between the two genotypes emerged in redox homeostasis, particularly L-ascorbate peroxidase, superoxide dismutase, and glutathione s-transferase, as well as higher accumulation of other stress-response proteins such as HSPs in the T genotype than the S genotype. Based on our results, it is likely that the T genotype has higher membrane integrity than the S genotype—as evidenced by its reduced LA and MDA content, higher RWC, and abundance of proteins such as heat- and cold-shock proteins, L-ascorbate peroxidase, superoxide dismutase, and glutathione s-transferase—which could reduce dehydration stress injuries to membranes and possibly increase water-use efficiency. Up-regulation of the auxin-binding protein after 1 d of stress, especially in the T genotype, suggests its involvement in the initial stages of the auxin signal transduction pathway and, in turn, its function as a possible integrator for other phytohormones to control plant growth under stress conditions. Overall, the results have offered insight into the proteome dynamics of drought tolerance in chickpea, and a framework for further functional studies on each identified protein.

## Supplementary information

**Additional file 1.**

## Data Availability

All the data generated or analyzed during this study are included in this published article.

## Abbreviations

ECM: Extracellular matrix
ROS: Reactive oxygen species
2-DE: Two-dimensional electrophoreses
MALDI-TOF/TOF: Matrix Assisted Laser Desorption Ionization - Time of Flight
LC-MS/MS: Liquid chromatography–mass spectrometry
LEA: Late embryogenesis abundant
HSPs: Heat shock proteins
TCA cycle: Tricarboxylic acid cycle
RWC: Relative water content
FW: Fresh weight
DW: Dry weight
TW: Turgid weight
EL: Electrolyte leakage
MDA: Malondialdehyde
TCA: Trichloroacetic acid
IEF: Isoelectric focusing
DTT: Dithiothreitol
IPG: Immobilized pH gradient
SDS: Sodium dodecyl sulfate
*M*_r_: Molecular mass
p*I*: Isoelectric point
ANOVA: Analysis of variance
DEPs: Differentially expressed proteins
PSI and PSII: Photosystem I and II
RuBisCo: Ribulose 1,5-bisphosphate carboxylase/oxygenase
SOD: Cu/Zn superoxide dismutase
