# Supplementary material for: Proteomic responses to progressive dehydration stress in leaves of chickpea seedlings
Source: BMC Genomics. 2020 Jul 29;21:523. doi: 10.1186/s12864-020-06930-2 (PMC7392671; doi:10.1186/s12864-020-06930-2)
Supplement: Supplementary file 1 — Additional file 1. [file 12864_2020_6930_MOESM1_ESM.docx]

Full-length gels


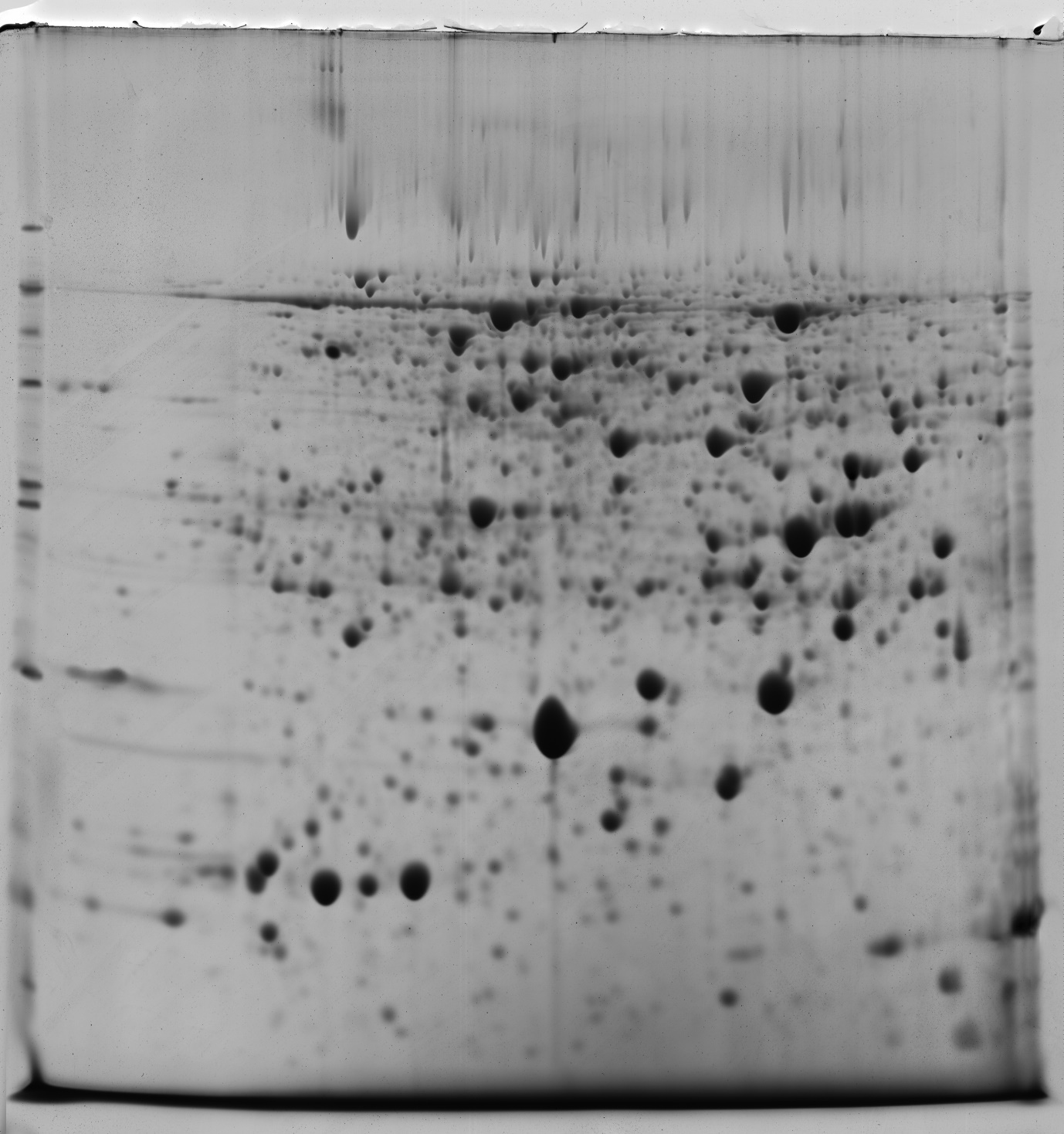


(A)


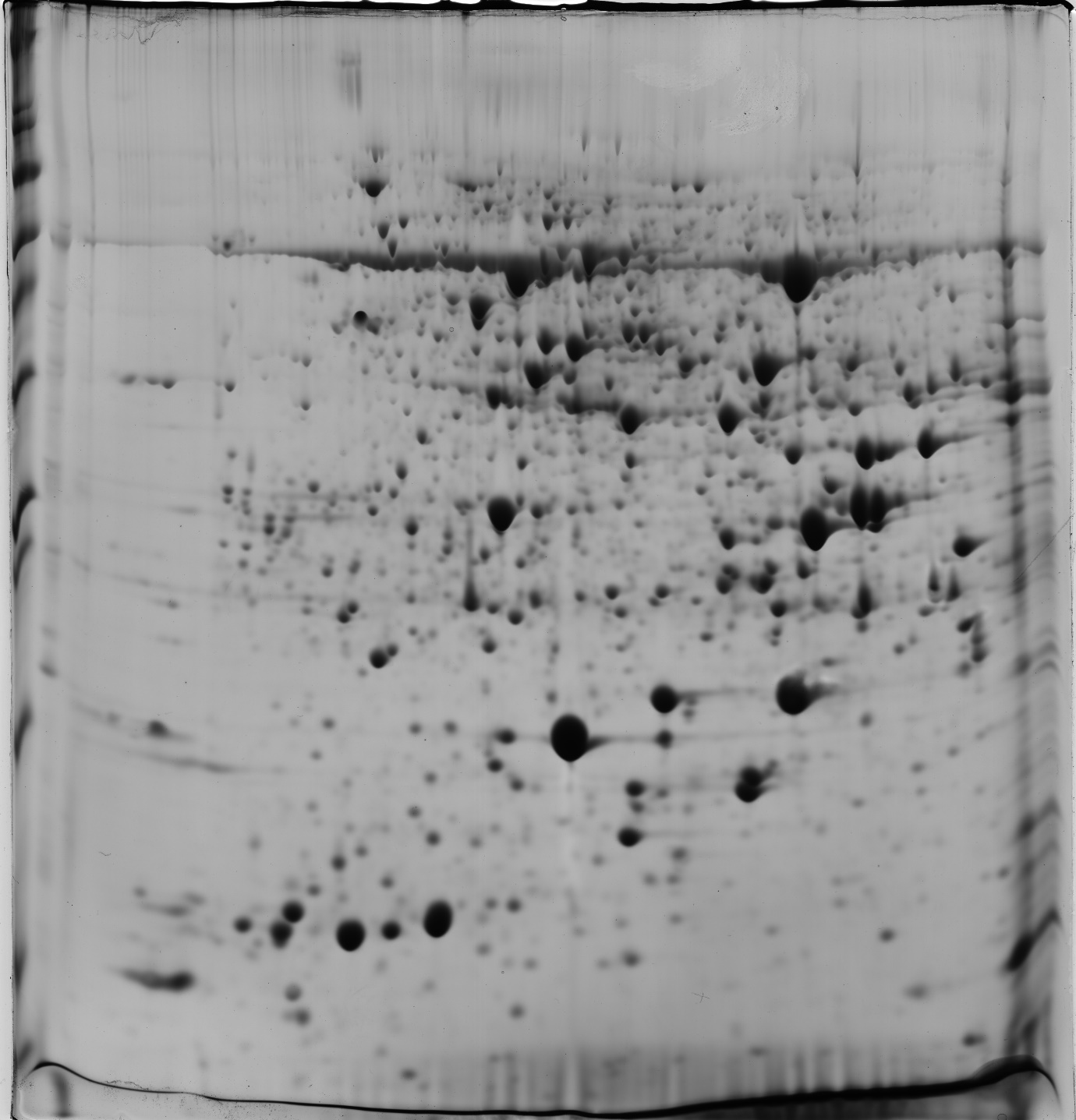


(B)

Representative 2-D photos of chickpea leaf proteins stained by Coomassie blue in the A. drought-tolerant (MCC537, T), and B. drought-sensitive (MCC806, S) genotypes. First dimension: 17 cm IEF strips pH 4–7 linear, second dimension: SDS-PAGE containing 12.5% (w/v) polyacrylamide. Lines indicate differentially regulated protein spots subjected to LC-MS/MS analysis.
